# Aspergillus spondylitis: case series and literature review

**DOI:** 10.1186/s12891-020-03582-x

**Published:** 2020-08-22

**Authors:** Guohua Dai, Ting Wang, Chuqiang Yin, Yuanliang Sun, Derong Xu, Zhongying Wang, Liangrui Luan, Jianwen Hou, Shuzhong Li

**Affiliations:** Department of Spine Surgery, Affiliated Hospital of Qingdao University, 16 jiangsu road, Shinan District, Qingdao City, Post Code: 266000 Shandong Province China

**Keywords:** Aspergillus spondylitis, Clinical features, Diagnosis and treatment, Prognosis

## Abstract

**Background:**

Spinal fungal infections, especially spinal Aspergillus infections, are rare in the clinic. Here, we introduce the clinical features, diagnosis, treatment, and prognoses of 6 cases of Aspergillus spondylitis.

**Methods:**

We retrospectively analysed the complete clinical data of patients with Aspergillus spondylitis treated in our hospital from January 2013 to January 2020.

**Results:**

Aspergillus fumigatus was isolated in 4 cases, and Aspergillus spp. and Aspergillus niger were isolated in 1 case each. All six patients reported varying degrees of focal spinal pain; one patient reported radiating pain, one patient experienced bowel dysfunction and numbness in both lower limbs, and three patients had fever symptoms. One case involved the thoracic spine, one case involved the thoracolumbar junction, and 4 cases involved the lumbar spine. Three patients were already in an immunosuppressed state, and three patients entered an immunosuppressed state after spinal surgery. All six patients were successfully cured, and five required surgery. Of the 5 patients who underwent surgical treatment, 2 had spinal cord compression symptoms, and 3 had spinal instability. At the end of follow-up, 1 patient reported left back pain and 1 patient reported left limb numbness.

**Conclusion:**

The clinical manifestations of Aspergillus spondylitis are non-specific, and the diagnosis depends on typical imaging findings and microbiological and histopathological examination results. When there is no spinal instability, spinal nerve compression symptoms, or progressive deterioration, antifungal therapy alone may be considered. If spinal instability, spinal nerve compression, or epidural abscess formation is present, surgery combined with antifungal therapy is recommended.

## Background

Aspergillus is a saprophytic fungus that mainly inhabits soil and plants [1]. Aspergillosis is an opportunistic infection that mainly affects the respiratory tract and can be spread through blood contact [2]. Severe neutropenia, glucocorticoid use, transplantation and other immunosuppressive conditions may lead to invasive infections [3]. Aspergillus infection can occur in most organs, but the lung is the main target organ. Among extrapulmonary infections, approximately 1.82% involve the skeletal muscle system [4], and of those, approximately half involve the spine [5–7]. Due to its potentially delayed onset and non-specific clinical manifestations, it is often misdiagnosed at initial presentation [8]. This study retrospectively analysed 6 Aspergillus spondylitis cases with complete clinical data in our hospital’s system from January 2013 to January 2020 and 66 cases Aspergillus spondylitis with relatively complete data from foreign literature.

## Methods

This study is a retrospective analysis of Aspergillus spondylitis. The 6 patients in this case series were admitted to the hospital for spinal surgery from 2013 to 2019. Our institution is a tertiary diagnosis and treatment centre that specializes in the diagnosis, treatment, and research of spinal diseases**.** The inclusion criteria were as follows: (1) patients with spinal infections with typical imaging characteristics and (2) patients with biopsy- and culture-confirmed Aspergillus infection. The exclusion criteria were as follows: (1) patients with other types of spinal infections and (2) patients with incomplete clinical data or who were lost to follow-up. Indications for surgery**:** In the case of spinal instability, spinal nerve compression, epidural abscess formation, or progressive deterioration, surgery combined with antifungal therapy is recommended.

Six patients, including 5 males and 1 female, were included in the case series. The patients were aged 43–68 years, with an average age of 57 years. This study did not exclude any cases. Five cases involved the lumbar spine and 1 case involved the thoracolumbar junction. All 6 patients had focal spinal pain of varying degrees, and 3 patients had fever symptoms. At admission, 2 patients had spinal nerve compression symptoms, 3 patients had spinal instability, and all underwent surgical treatment. Only 1 patient had no indication for surgery and was cured after conservative treatment. The course of the disease ranged from 25 days to 5 years. The treatment time ranged from 4 to 6 months, with an average of 4.5 months. The follow-up time ranged from 15 to 24 months, with an average of 22 months. See Tables 1 and 2.

**Table 1:** Clinical and laboratory characteristics of the 6 patients in this case series

Cases	Age	Sex	Symptom	Species	Level	CRP (mg/L)	ESR (mm/h)	(Fungal G test for 1–3-β-D glucan (pg/mL),100.5–151.5: the early infection; <100.5:-; >151.5:+	(GM test for Aspergillus) Immunological test for Aspergillus(ug/L); <0.65:-; ≥ 0.85:+; 0.65–0.85:±
1	67	Male	Back pain with radiating pain in the bilateral rib area	*A. fumigatus*	T3–5	9.22	66	1744.3(+)	0.66(±)
2 (presentation)	68	Male	Back pain, urinary retention, numbness and weakness in the limbs	*A. fumigatus*	T12-L2	44.89	34	181.4(+)	0.37(−)
3	50	Female	Back pain, fever	*A. fumigatus*	L3–4	–	115	–	–
4	48	Male	Back pain	*A. fumigatus*	L4–5	–	23	62.43(−)	–
5	43	Male	Back pain, fever	*A. niger*	L4–5	34.88	32	–	–
6	66	Male	Back pain, fever	*Aspergillus* spp.	L2–3	15.74	45	–	–

**Table 2:** Treatment and prognosis of the 6 patients in this case series

Case	Risk factors	Surgery	Antifungal treatment	Outcome	Treatment time (months)	Follow -up period (months)
1	Lung cancer surgery, pulmonary aspergillosis	No, needle biopsy	Voriconazole,200 mg, every 12 h; 20 weeks	Cured	4	20
**2 (presentation)**	Steroid use	Laminectomy, Debridement, decompression, Instrumentation, (posterior)	Voriconazole, 200 mg, every 12 h; 16 weeks	Cured, remaining left limb numbness	4	24
3	Renal failure, haemodialysis	Laminectomy, Debridement, Instrumentation, (posterior)	Voriconazole, 200 mg, every 12 h; 18 weeks	Cured, remaining lumbar pain	6	24
4	History of minimally invasive spinal surgery	Laminectomy, Debridement, Instrumentation, (posterior)	Voriconazole, 200 mg, every 12 h; 16 weeks	Cures	4	15
5	Spine surgery	Laminectomy, Debridement, decompression, Instrumentation, (posterior)	Voriconazole, 200 mg, every 12 h; 22 weeks	Cured	5	20
6	Spine surgery	Laminectomy, Debridement, Instrumentation, (posterior)	Voriconazole, 200 mg, every 12 h; 20 weeks	Cured	4	18

Detailed data regarding clinical symptoms (focal spine pain, neurological symptoms, fever, etc.), serological examinations (white blood cell (WBC) count, erythrocyte sedimentation rate (ESR), C-reactive protein (CRP)), laboratory examinations (fungal G test for (1–3)-β-D glucan, Aspergillus galactomannan (GM) test), microbiological examinations (bacterial culture, fungal culture), imaging examinations (typical low T1WI signal, high T2WI signal on MRI), histopathological examinations, biopsy findings (hexamine silver staining (GMS), periodic acid-colourless magenta (PAS) staining) and other examinations were collected to analyse the clinical features, diagnosis, treatment, and prognosis of Aspergillus spondylitis.

### Treatment

**Fig. 1:**
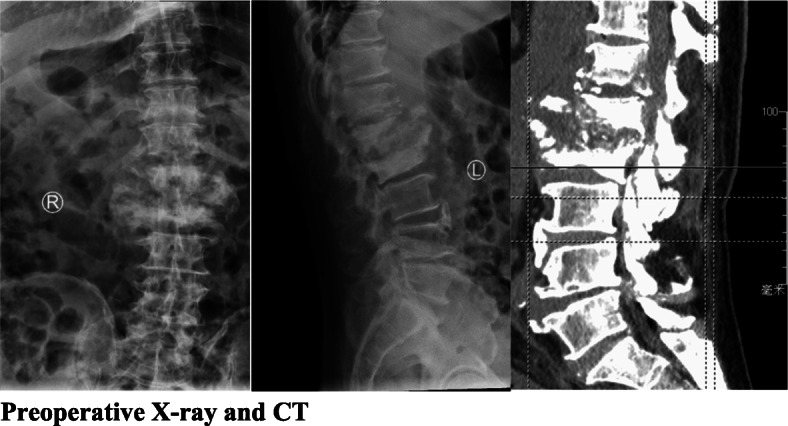
Preoperative X-ray and CT

**Fig. 2:**
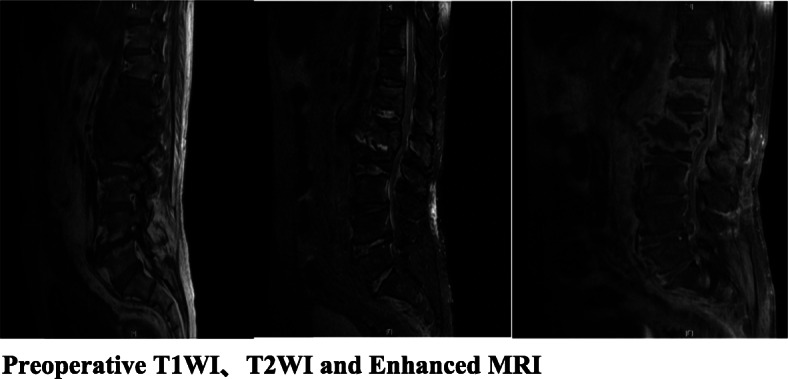
Preoperative T1WI、T2WI and Enhanced MRI

**Fig. 3:**
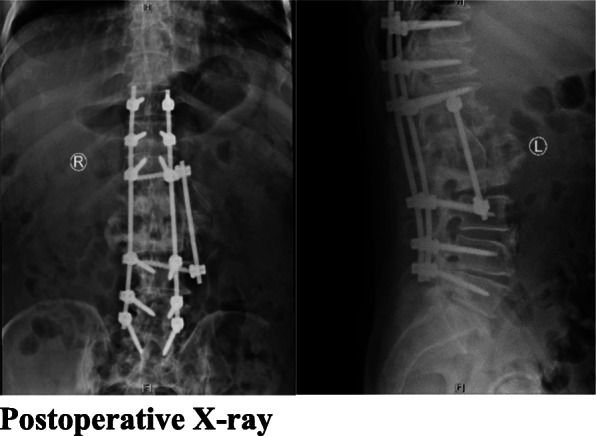
Postoperative X-ray

**Fig. 4:**
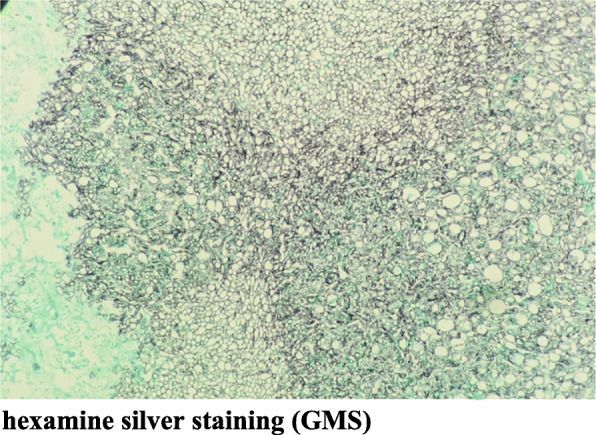
hexamine silver staining (GMS)

All patients underwent WBC counts; ESR analyses X-ray, computed tomography (CT), and magnetic resonance imaging (MRI) examinations; tissue bacterial culture, and histopathological examinations. Among the 6 patients, 2 patients (2/6) had elevated WBC counts, 6 patients (6/6) had elevated ESRs, and 4 patients (4/5) had elevated CRP. Three patients (1–3) were positive for (1–3)-β-D glucan on the fungal G test, and two patients (2/3) were positive for Aspergillus GM on the Aspergillus GM test. Six patients (6/6) had positive tissue cultures, and 3 patients (3/6) had observable fungal hyphae. The results of bacterial culture were 4 cases of *Aspergillus fumigatus*, 1 case of *Aspergillus niger* and 1 case of *Aspergillus* spp. One patient underwent CT-guided puncture of the intervertebral disc and vertebral body tissue. Samples underwent histopathological examination and were positive according to the hexamine silver staining (GMS) method and the periodate-colourless magenta (PAS) method, and fungal hyphae were visible under the microscope (Aspergillus). In 5 patients, intervertebral disc and vertebral body tissues were sampled and sent for histopathological examination. Samples were positive according to the GMS method and (PAS) method, and fungal hyphae were visible under the microscope (Aspergillus) (Tables 1, 2, 3 and Figs. 1, 2, 3, and 4).

**Table 3:** Aspergillus classification: 66 cases in the literature, 6 cases in this series

Species	Value	Value
*A. fumigatus*	47	4
*A. flavus*	5	
*A. niger*	1	1
*A. terreus*	3	
*A. nidulans*	4	
*A. versicolour*	1	
*Aspergillus* spp.	5	1

#### Statistical analysis

This study did not employ statistical analysis.

## Results

All six patients were successfully cured; five required surgery. Of the 5 patients who underwent surgical treatment, 2 had spinal cord compression symptoms, and 3 had spinal instability. The antifungal drug administered was voriconazole. At the end of the follow-up period, 1 patient reported left back pain, and 1 patient experienced left lower limb numbness. The follow-up time varied from 15 to 24 months, with an average of 22 months. See Tables 1, 2, and 3.

## Discussion

Aspergillus spondylitis is a rare opportunistic fungal infectious disease; however, in recent years, its prevalence has surpassed that of Candida spondylitis [9]. Most patients suffer from impaired immune function or chronic diseases (due to AIDS, organ transplantation, chemotherapy or immunosuppressive therapy, etc.] [2, 10]; however, patients with normal immune function but predisposing factors (pulmonary aspergillosis, tuberculosis, discectomy, diabetes, chronic obstructive pulmonary disease, fungal heart disease, endometritis, etc.) may also be affected [11, 12]. The pathogenesis of Aspergillus spondylitis includes continuous spread to adjacent lung foci, exposure to contaminated blood, and direct inoculation (during trauma or surgery) [6]. Most of the cases in this case series and all 66 cases in the literature were transmitted by blood exposure.

Aspergillus spondylitis lacks specific clinical features. The most common is lower back pain with or without fever. When an epidural abscess forms, symptoms of spinal nerve compression and even paraplegia may occur. Men are affected more frequently than women, and most infections involve the lumbar spine, followed by the thoracic spine and cervical spine. In this case series and in cases in the literature, lower back pain and numbness in the lower extremities were the main clinical manifestations. All 6 patients in this group experienced lower back pain; 2 also had neurological symptoms, and 3 had fever. Of the 66 patients in the literature, 56 had lower back pain, 27 had neurological dysfunction, and 25 had fever. Its clinical characteristics are consistent with those reported in the literature [8].

To confirm the diagnosis, a variety of diagnostic tools, including routine fungal culture and unconventional microbiological testing, such as antibody/antigen and molecular testing, as well as histopathology and radiology examinations, must be combined [13]. WBC counts have poor sensitivity and specificity and are of little value for diagnosis. Normal levels of inflammatory markers (ESR, CRP, etc.) do not rule out Aspergillus infection because immunocompromised patients cannot produce a significant inflammatory response, but regular monitoring of inflammatory marker levels may help to assess response to treatment [14]. Studies [15] indicate that the fungal G test for (1–3)-β-D-glucan and the Aspergillus GM test are meaningful for the diagnosis of mycotic spondylitis. Among the 66 cases in the literature, 47% had elevated WBC counts. The ESR was increased by 92%, and CRP was increased by 75%. In the current case series, 2 patients (2/6) had elevated WBC counts, all 6 patients (6/6) had elevated ESRs, and 4 patients (4/5) had elevated CRP.

The imaging manifestations of Aspergillus spondylitis are related to the patient’s immune function and course of disease [11, 16]. X-ray and CT manifestations are often atypical. The typical manifestations on MRI are a low T1WI signal, an iso-signal or a slightly high T2WI signal, a high lipid-suppressing sequence signal, and obvious enhancement. Fifty-six cases in the literature had typical imaging findings; 5 patients in current case series had typical imaging findings, and 1 patient was misdiagnosed with Brucella spondylitis on admission. Brucellosis spondylitis is associated with multi-vertebral imaging features. In addition to distinguishing it from physical and jumping injuries, attention should also be paid to distinguish it from other infections (other fungal infections, spinal tuberculosis, suppurative spondylitis, etc.) [17, 18].

The most reliable diagnostic methods for Aspergillus spondylitis are histopathological examination and bacterial culture. When haematological (ESR, CRP, fungal G test, etc.) and imaging examinations indicate Aspergillus spondylitis, CT-guided puncture biopsy should be performed as soon as possible to confirm the diagnosis and guide antibacterial treatment [19]. Compared with intraoperative biopsy, CT-guided biopsy is less invasive and has a quicker recovery time, but the number of specimens is insufficient. In addition, blood culture should also be routinely performed, combining multiple diagnostic methods. Although the positive rate of blood culture is low, it can guide the selection of antifungal drugs when tissue culture is not possible.

Compared with amphotericin B preparations, broad-spectrum triazoles [20] are often better tolerated, and the survival rate in patients is improved, especially in invasive fungal infection patients. According to the Aspergillosis Treatment Guidelines [21] proposed by the American Society of Infectious Diseases 2016 (the latest version), voriconazole is recommended as the main treatment for invasive aspergillosis, including Aspergillus osteomyelitis. For refractory patients that cannot tolerate conventional antifungal treatment, itraconazole is recommended as an alternative therapy for invasive aspergillosis [22]. A large randomized controlled trial [23] demonstrated the superiority of voriconazole, including improved survival rates and reduced toxicity, over AmB in the treatment of invasive aspergillosis. A recent study [24] showed that voriconazole achieved better results than AmB. Voriconazole is associated with a higher survival rate than AmB in fungal infection patients, and invasive aspergillosis treated with voriconazole has a higher remission rate than that treated with AmB. In the literature, 70% of patients were treated with triazoles, 30% were treated with AmB, and 42 cases were cured; all 6 patients in the current case series were treated with voriconazole antifungal therapy, and all were cured.

When nerve and spinal cord compression, spinal instability, or epidural abscess formation is present, conservative antifungal treatment is not effective and can even progressively worsen the disease prognosis; thus, surgery combined with antifungal treatment should be considered [21, 25, 26]. Of the 66 cases in the literature, 44 cases (67%) received combined surgical and antifungal therapy, 22 cases were treated with antifungal therapy alone, and 42 cases were cured. Five patients in the current case series underwent received combined surgical and antifungal therapy, and one patient received antifungal therapy alone; all of the patients were cured.

The prognoses in the current case series and those in the literature are shown in Table 4. A poor prognosis may be due to a patient’s low immune function or poor overall health. A patient’s response to and outcome of treatment largely depend on host factors [27], including neurological diseases, underlying diseases, and early diagnosis and treatment.

**Table 4:** Clinical characteristics of Aspergillus spondylodiscitis: 66 cases in the literature, 6 cases in this series

Features	Cases in the literature	Cases in this series
Age		
Range	4–76	43–68
Mean age	43	57
≥ 18	58	6
<18	8	0
Male	45	5
Female	21	1
Symptoms		
Back pain	56	6
Neurological deficits	27	1
Level		
Cervical	5	0
Thoracic	29	1
Lumbar or sacral	35	5
WBC > (10 × 10^9^/L)	22 of 47 (46.8%)	2 of 6(33.3%)
ESR > (20 mm/h)	24of 27(91.9%)	6 of 6(100%)
CRP > (5 mg/L)	12 of 16(75%)	4 of 4(100%)
Surgery	44	5
Relapse	4	0
Cure	42	6
Sequelae	5	2
Death	16	0

## Conclusions

The clinical manifestations of Aspergillus spondylitis are non-specific, and the diagnosis depends on typical imaging findings and microbiological and histopathological examination results. When spinal instability, spinal nerve compression symptoms, and progressive deterioration are absent, antifungal therapy alone may be considered. When spinal instability, spinal nerve compression, or epidural abscess formation is present, surgery combined with antifungal therapy is recommended.

This study has certain limitations. First, this was a retrospective case series, limited by the inherent flaws of the retrospective design. Second, the small sample size prevented us from performing a descriptive analysis. Finally, the follow-up time limited the evaluation of final patient outcomes.

## Data Availability

The datasets used and analysed during the current study are available from the corresponding author on reasonable request.

## Abbreviations

WBC: white blood cell
ESR: erythrocyte sedimentation rate
CRP: C-reactive protein
T1WI: T1 Weighted Image
T2WI: T2 Weighted Image
CT: Computed tomography
MRI: Magnetic Resonance Imaging
AmB: amphotericin B
GMS: Silver hexamine stain
PAS: periodic acid schiff

## References

[1] Hébert-Seropian S, Pelet S (2020). Aspergillus osteomyelitis of the scapula: A case report. JBJS Case Connector.

[2] Senosain-Leon V, Hidalgo-Benites A, Arriola-Montenegro J (2019). Invasive pulmonary aspergillosis with Aspergillus vertebral osteomyelitis in an HIV-infected adult: a case report. Int J STD AIDS.

[3] Henry MW, Miller AO, Walsh TJ (2017). Fungal musculoskeletal infections. Infect Dis Clin N Am.

[4] Takagi Y, Yamada H, Ebara H (2019). Aspergillus terreus spondylodiscitis following an abdominal stab wound: a case report. J Med Case Rep.

[5] Gabrielli E, Fothergill AW, Brescini L (2014). Osteomyelitis caused by Aspergillus species: a review of 310 reported cases. Clin Microbiol Infect.

[6] Gamaletsou MN, Rammaert B, Bueno MA (2014). Aspergillus osteomyelitis: epidemiology, clinical manifestations, management, and outcome. J Inf Secur.

[7] Koehler P, Tacke D, Cornely OA (2014). Aspergillosis of bones and joints - a review from 2002 until today. Mycoses.

[8] Jiang Z, Wang Y, Jiang Y (2013). Vertebral osteomyelitis and epidural abscess due to Aspergillus nidulans resulting in spinal cord compression: case report and literature review. J Int Med Res.

[9] Ganesh D, Gottlieb J, Chan S (2015). Fungal infections of the spine. Spine (Phila Pa 1976).

[10] Vinas FC, King PK, Diaz FG (1999). Spinal aspergillus osteomyelitis. Clin Infect Dis.

[11] Nicolle A, De La Blanchardière A, Bonhomme J (2013). Aspergillus vertebral osteomyelitis in immunocompetent subjects: case report and review of the literature. Infection.

[12] Comacle P, LE Govic Y, Hoche-Delchet C (2016). Spondylodiscitis due to Aspergillus terreus in an Immunocompetent host: case report and literature review. Mycopathologia.

[13] Ruhnke M, Behre G, Buchheidt D (2018). Diagnosis of invasive fungal diseases in haematology and oncology: 2018 update of the recommendations of the infectious diseases working party of the German society for hematology and medical oncology (AGIHO). Mycoses.

[14] Gupta PK, Mahapatra AK, Gaind R (2001). Aspergillus spinal epidural abscess. Pediatr Neurosurg.

[15] Su KC, Chou KT, Hsiao YH (2017). Measuring (1,3)-β-D-glucan in tracheal aspirate, bronchoalveolar lavage fluid, and serum for detection of suspected Candida pneumonia in immunocompromised and critically ill patients: a prospective observational study. BMC Infect Dis.

[16] Yoon KW, Kim YJ (2015). Lumbar Aspergillus osteomyelitis mimicking pyogenic osteomyelitis in an immunocompetent adult. Br J Neurosurg.

[17] Saeed K, Esposito S, Ascione T (2019). Hot topics on vertebral osteomyelitis from the International Society of Antimicrobial Chemotherapy. Int J Antimicrob Agents.

[18] Orlowski HLP, Mcwilliams S, Mellnick VM (2017). Imaging Spectrum of invasive fungal and fungal-like infections. Radiographics.

[19] Wu CJ, Liu WL, Lai CC (2020). Multicenter study of azole-resistant Aspergillus fumigatus clinical isolates, Taiwan(1). Emerg Infect Dis.

[20] Jenks JD, Mehta SR, Hoenigl M (2019). Broad spectrum triazoles for invasive mould infections in adults: Which drug and when?. Med Mycol.

[21] Patterson TF, Thompson GR, Denning DW (2016). Practice Guidelines for the Diagnosis and Management of Aspergillosis: 2016 Update by the Infectious Diseases Society of America. Clin Infect Dis.

[22] Batra S, Arora S, Meshram H (2011). A rare etiology of cauda equina syndrome. J Infection Developing Countries.

[23] Herbrecht R, Denning DW, Patterson TF (2002). Voriconazole versus amphotericin B for primary therapy of invasive Aspergillosis [J]. N Engl J Med.

[24] Herbrecht R, Patterson TF, Slavin MA (2014). Application of the 2008 definitions for invasive fungal diseases to the trial comparing Voriconazole versus amphotericin B for therapy of invasive Aspergillosis: A collaborative study of the mycoses study group (MSG 05) and the European Organization for Research and Treatment of Cancer infectious diseases group. Clin Infect Dis.

[25] Garciavidal C, Alastrueyizquierdo A, Aguilarguisado M, et al. Executive summary of clinical practice guideline for the management of invasive diseases caused by Aspergillus: 2018 Update by the GEMICOMED-SEIMC/REIPI. Enfermedaders infecciosasy microbiologia clinica. 2019;37(8):535–41. 10.1016/j.eimc.2018.03.018 29960829

[26] Yang H, Shah A A, Nelson S B, et al. Fungal spinal epidural abscess: a case series of nine patients. Spine J. 2019;19(3):516–22. 10.1016/j.spinee.2018.08.001 30121322

[27] Bassetti M, Peghin M, Vena A (2018). Challenges and solution of invasive Aspergillosis in non-neutropenic patients: A review. Infect Dis Ther.

